# Elevating Detection Performance in Optical Remote Sensing Image Object Detection: A Dual Strategy with Spatially Adaptive Angle-Aware Networks and Edge-Aware Skewed Bounding Box Loss Function

**DOI:** 10.3390/s24165342

**Published:** 2024-08-18

**Authors:** Zexin Yan, Jie Fan, Zhongbo Li, Yongqiang Xie

**Affiliations:** Institute of System Engineering, Academy of Military Sciences, Beijing 100141, China; yanzexin@outlook.com (Z.Y.); hanyovladscarlet@mail.nwpu.edu.cn (J.F.)

**Keywords:** optical remote sensing, object detection, discontinuous boundary, rotation equivariant

## Abstract

In optical remote sensing image object detection, discontinuous boundaries often limit detection accuracy, particularly at high Intersection over Union (IoU) thresholds. This paper addresses this issue by proposing the Spatial Adaptive Angle-Aware (${\mathrm{SA}}^{3}$) Network. The ${\mathrm{SA}}^{3}$ Network employs a hierarchical refinement approach, consisting of coarse regression, fine regression, and precise tuning, to optimize the angle parameters of rotated bounding boxes. It adapts to specific task scenarios using either class-aware or class-agnostic strategies. Experimental results demonstrate its effectiveness in significantly improving detection accuracy at high IoU thresholds. Additionally, we introduce a Gaussian transform-based IoU factor during angle regression loss calculation, leading to the development of Edge-aware Skewed Bounding Box Loss (EAS Loss). The EAS loss enhances the loss gradient at the final stage of angle regression for bounding boxes, addressing the challenge of further learning when the predicted box angle closely aligns with the real target box angle. This results in increased training efficiency and better alignment between training and evaluation metrics. Experimental results show that the proposed method substantially enhances the detection accuracy of ReDet and ReBiDet models. The ${\mathrm{SA}}^{3}$ Network and EAS loss not only elevate the mAP of the ReBiDet model on DOTA-v1.5 to 78.85% but also effectively improve the model’s mAP under high IoU threshold conditions.

## 1. Introduction

In the field of optical remote sensing image target detection, the random orientation of almost all objects in the image often leads to horizontal bounding boxes encompassing a significant amount of irrelevant background when annotating detected objects. In comparison, rotated bounding boxes can greatly improve the pixel area ratio between objects and background within the selected region, better accommodating the annotation of targets in arbitrary directions. They provide accurate information about the direction, position, and size of the targets, enabling the model to perform better in target detection tasks. Existing methods for rotated object detection are mainly built upon generic object detection approaches. They redefine the representation of detection boxes, introduce additional angular dimensions for rotated detection boxes, and optimize them through distance loss. Rotated object detection methods have played a crucial role in various fields, including text detection [1,2,3,4,5,6], face recognition [7,8,9], and remote sensing image target detection [10,11,12].

However, introducing angle parameters also introduces uncertainty into the detection task, with the most prominent issue being the discontinuous boundary problem. Additionally, because the evaluation metric for the final model is based on IoU values rather than angular differences, optimizing the predicted box angles through distance loss introduces a mismatch between loss calculation and evaluation metrics. These two issues have led to suboptimal performance of existing models under high IoU threshold conditions (IoU > 0.5). Achieving higher accuracy in rotated object detection has become a significant research focus.

This section first introduces the discontinuous boundary problem, describing the origins of the discontinuity in parameterized regression. It then discusses the mismatch between the calculation of angles in the loss function and evaluation metrics. Following that, it surveys the progress of domestic and international research and concludes by introducing the research objectives of this paper.

### 1.1. Discontinuous Boundary Issue

Rotated bounding boxes are commonly represented in two ways: the five-parameter representation [1,5,11,12,13,14,15] and the eight-parameter representation [2,16,17,18]. Both representations can describe the position, size, and rotation angle of a rotated bounding box. The five-parameter representation is often preferred due to its simplicity, intuitive nature, and the reduced number of parameters for computation and storage. In this representation, scholars have devised various methods for representing rotated boxes, establishing a range for the angle in radians, binding it closely with the height and width of the detection box to avoid the confusion of multiple angle values caused by the angular periodicity.

Due to different definitions of the θ range, three major categories of rotated box definitions have gradually emerged in rotated object detection. The OpenCV definition [5,12,13,14,15], abbreviated as OC, sets the angle range as angle $\in (0,90^{\circ }]$, $\theta \in (0,\frac{1}{2}\pi ]$, where the acute angle between the positive x-axis and the edge defined as the width is positive. The long edge definition [5,11] defines the long edge as the width and the short edge as the height. The angle θ is the angle between the long edge width and the x-axis. Based on the angle range, it is further divided into le135 and le90 definitions. The angle ranges are set as $\mathrm{angle}\in [-45^{\circ },135^{\circ })$ or $\theta \in [-\frac{\pi }{4},\frac{3\pi }{4})$ for le135, and $\mathrm{angle}\in [-90^{\circ },90^{\circ })$ or $\theta \in [-\frac{\pi }{2},\frac{\pi }{2})$ for le90.

**Figure 1:**
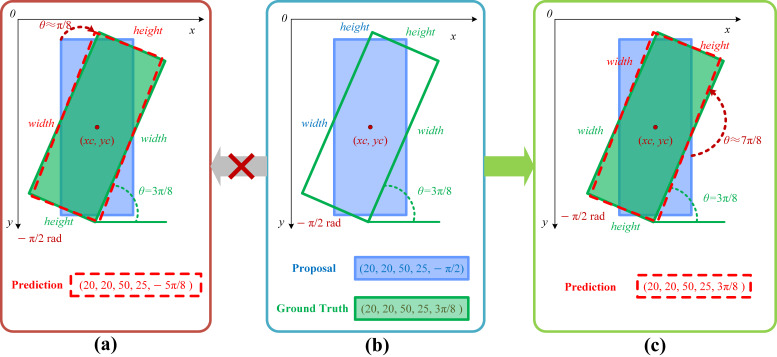
Prediction box generation method under le90 definition and CCW representation conditions. (**a**) Ideal regression path for generating prediction boxes. (**b**) Initial position of the proposal. (**c**) Actual regression path for generating prediction boxes.

In parameterized regression-based rotational detection methods, as illustrated in Figure 1, significant differences exist between the ideal regression path and the actual regression path under the le90 definition and the counter-clockwise (CCW) representation. When regressing candidate boxes, if the ideal regression path (indicated by the dark red dashed arrow in Figure 1a) is followed, the candidate box rotates clockwise by $\frac{1}{8}\pi$ to approximate the true target box. Although the predicted box has an IoU≈1 with the true target box, the loss value is significantly greater than 0. This is because the angle of the predicted box changes from $-\frac{1}{2}\pi$ to $-\frac{5}{8}$, increasing the discrepancy with the true target box’s angle of $\frac{3}{8}\pi$. Due to the periodic nature of angles, although the boxes are almost coincident visually, the angle difference leads to an increase in the loss value. In this situation, the model needs to regress along a more distant path, namely, counter-clockwise rotation by $\frac{7}{8}\pi$ (indicated by the dashed red arrow in Figure 1c), achieving an IoU≈1 and a loss value Loss≈0 to generate the final predicted box.

In summary, the discontinuous boundary problem is primarily caused by the angular periodicity and the exchangeability of the long and short sides in the five-parameter representation. The ideal regression path exceeds the predefined range, encountering boundary issues, leading to an increase in regression loss.

Faced with this challenge, the transformation of predicted boxes under the OpenCV definition involves both the exchange of width and height, as well as angle transformation, resulting in an overall complexity that may lead to less accurate predictions in such conditions [19]. In contrast, the long edge definition only necessitates considering a broad range of angle transformations when addressing the issue of discontinuous boundaries. To align with the module design in the subsequent sections of this article, the le90 definition is adopted as the angle range definition for rotated bounding boxes. Even in the case of the four-point representation method, discontinuous boundary problems may arise due to the order of corner points, a matter which will not be further elaborated upon here.

### 1.2. Mismatch between Loss and Evaluation Metrics

Over the years, the primary approach to improving the detection accuracy of rotated object detection models has been to propose better network architectures and more effective strategies for extracting reliable local features. However, there is a largely overlooked optimization path that has received limited research attention in the field: designing a novel loss function to replace the commonly used regression loss function, aiming to enhance the model’s detection performance. In object detection, the Intersection over Union (IoU) between the detection boxes generated by the model and the ground truth boxes is a crucial evaluation metric used to calculate the mAP. Therefore, in rotated object detection, special consideration needs to be given to minimizing angle errors and maximizing IoU values.

**Figure 2:**
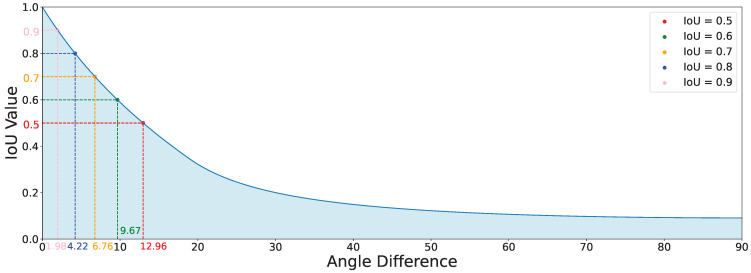
Relationship curve between angular difference and IoU for rectangles with a 1:6 aspect ratio and overlapping centroids.

Most existing state-of-the-art rotated object detection models largely adopt the widely used Smooth L1 loss [20,21] as the loss function for the regression branch [22,23], following common practice in general object detection. This loss calculates the value based on the numerical differences between two bounding boxes to minimize coordinate, aspect ratio, and angle deviations. As depicted in Figure 2, the curve illustrates that the angle difference between two rectangles with an aspect ratio of 1:6 and overlapping centroids does not show a linear relationship with IoU values. When the angle difference is less than 20°, a smaller angle difference results in a larger change in IoU. Specifically, when the angle difference is within 12.96°, further reduction in the angle difference leads to a sharp increase in IoU values. However, at this point, the loss value of the model’s angle difference diminishes linearly. This scenario causes the model to lack sufficient loss gradient when the predicted box’s angle is close to the angle of the real target box. As a result, the model fails to learn how to generate more accurately angled detection boxes, preventing the detection boxes from further aligning accurately, which is clearly not an ideal outcome.

In summary, the Smooth L1 loss is not sufficiently sensitive to small angle errors, and a good local optimum based on the Smooth L1 loss may not necessarily be a local optimum for IoU. This makes it challenging to effectively guide the model to maximize the IoU values between the detection boxes and the real target boxes.

### 1.3. Related Work

Due to the complexity of scenes in remote sensing images, including small sizes, large aspect ratios, randomly and densely distributed objects, and arbitrarily oriented objects, rotation object detection models based on bounding box regression continue to dominate due to their higher accuracy and robustness. Parameterized regression methods are mainly divided into two branches.

**Single-stage:** DAL [24] proposes dynamic adaptive learning to dynamically select high-quality anchor boxes for accurate object detection, significantly reducing the number of predefined anchor boxes. R3Det [12] introduces a feature refinement module based on RetinaNet, using a progressive regression approach for fast and accurate object detection. It also suggests a skewed IoU (SkewIoU) loss to mitigate sensitivity to angle transformations in targets with large aspect ratios. RSDet [17] regresses rotated bounding boxes using four points and introduces a modulation rotation loss to address the discontinuity issue in existing losses when facing discontinuous boundaries. S2A-Net [25] introduces a Feature Alignment Module (FAM) for adaptive alignment with high-quality anchor boxes and an Orientation-aware Detection Module (ODM) for encoding orientation information, alleviating inconsistencies between the classification and regression branches. CSL [26] transforms angle prediction from a regression problem into a classification task to address the issue of discontinuous boundaries.

**Two-stage:** ICN [10] designs a joint image cascade network to extract multiscale semantic features and optimize regression losses. RoI Transformer [11] predicts a coarse rotated RoI based on RPN in its first stage and refines the prediction using RoI Align in the second stage, resulting in more accurate rotated RoIs and improving both efficiency and accuracy compared to rotated RPN. SCRDet [13] uses a sampling fusion network to enhance sensitivity to small objects and proposes supervised pixel attention networks and channel attention networks for joint detection of small and cluttered objects. It combines Smooth L1 loss with IoU factors to address discontinuous boundary issues in rotated bounding boxes. Gliding Vertex [18] accurately describes target orientation by sliding along each corresponding edge of a horizontal bounding box, introducing an area ratio factor between the target and its horizontal bounding box to guide the model in predicting quadrilateral detection boxes accurately. Oriented R-CNN [22] introduces a midpoint offset representation on Faster R-CNN, modifying the output parameters of the RPN regression branch from 4 to 6 to achieve rotated candidate boxes, significantly improving detection accuracy and computational efficiency. These are mainstream state-of-the-art two-stage detection models proposed in the last three years, inheriting the architecture of Faster R-CNN [21]. ReDet [23] introduces the concept of E(2)-Equivariant Steerable CNNs [27] (E(2)-CNNs) into object detection based on RoI Transformer, utilizing E2-CNNs to rewrite ResNet50 as ReResNet50. It redesigns the Rotation-invariant Region of Interest Align (RiRoIAlign) module, aligning in both channel and spatial dimensions to obtain rotation-invariant features. ReBiDet [28], based on ReDet, enhances feature fusion through the ReBiFPN module, balances the difficulty and proportion of positive samples during training using the DPRL module, and optimizes anchor box sizes and aspect ratios to further improve the model’s detection performance.

Existing object detection methods more or less involve the study of discontinuous boundary issues and mismatches between loss calculation and evaluation metrics. However, researchers have primarily concentrated on improving detection accuracy at IoU = 0.5, with limited attention to enhancing accuracy at IoU = 0.55 and above. These two issues result in suboptimal performance of current models at high IoU thresholds (greater than 0.5). As detection accuracy at IoU = 0.5 approaches a bottleneck, achieving higher precision in rotational object detection is poised to become a key focus of future research.

### 1.4. Goal of the Research

This paper addresses two primary issues: the discontinuous boundary problem and the mismatch between loss calculation and evaluation metrics. Solutions are proposed based on the ReBiDet model, and our work is organized into the following two aspects:

#### 1.4.1. Discontinuous Boundary Issue

We conduct a thorough analysis of the discontinuous boundary problem, focusing on its origins in parameterized regression. Our analysis reveals that the core issue is the inaccurate localization of detection boxes due to large-angle variations during the regression of rotated bounding boxes. To address this problem, we propose the Spatially Adaptive Angle-Aware (${\mathrm{SA}}^{3}$) Network, a cascaded structure designed to handle the large-angle variations caused by discontinuous boundaries. This network enhances the model’s ability to adapt to complex scenarios, thereby improving the accuracy of object detection.

#### 1.4.2. Mismatch between Loss Calculation and Evaluation Metrics

To tackle the issue of traditional loss functions being insensitive to Intersection over Union (IoU) values, we propose the Edge-aware Skewed Bounding Box Loss (EAS Loss). This novel loss function addresses the nonlinear decay of the loss value when the predicted box’s angle closely matches the target box’s angle. By incorporating this loss function, we aim to improve the alignment between the loss calculation and IoU values, enhancing overall model performance.

Additionally, we perform ablation studies and extensive comparative experiments with state-of-the-art models on two datasets to validate the effectiveness of the proposed modules and approaches.

## 2. Methods

### 2.1. Spatially Adaptive Angle-Aware Network

#### 2.1.1. Spatially Adaptive Angle-Aware Network Structure

The discontinuous boundary issue fundamentally results in imprecise localization of the final detection box due to the wide range of angle variations during rotated bounding box regression. Initial rotated object detection models, such as those based on Faster R-CNN [21], incorporated an angle parameter during the bounding box regression stage. However, the precision of the prediction boxes generated by a single regression was unsatisfactory. Even in general object detection, studies have shown that when dealing with candidate boxes of varying quality generated by the RPN module, a single regression function struggles to align all predicted bounding boxes accurately with the ground truth. Subsequently, the RoI Transformer [11] proposed a two-stage regression approach to produce more accurate prediction boxes. This strategy performed well under the condition of IoU = 0.5 and has been widely accepted by researchers, continuing to be adopted by state-of-the-art rotated object detection models. However, in rotated object detection, the introduction of the parameter θ exacerbates the discontinuous boundary issue. As a result, even with two regression functions, it remains challenging to achieve precise regression tasks in rotated object detection when IoU > 0.5.

Inspired by the Cascade R-CNN [29] approach, this subsubsection decomposes the regression task for rotated bounding boxes into three stages. Specifically, three dedicated regression functions $\left\{f_{1},f_{2},f_{3}\right\}$ are designed for each stage. These regression functions optimize the bounding boxes generated at their respective stages. The overall regression equation is as follows:

(1) $$F\left(x,b\right)=f_{3}\circ f_{2}\circ f_{1}\left(x,b\right),$$

**Figure 3:**
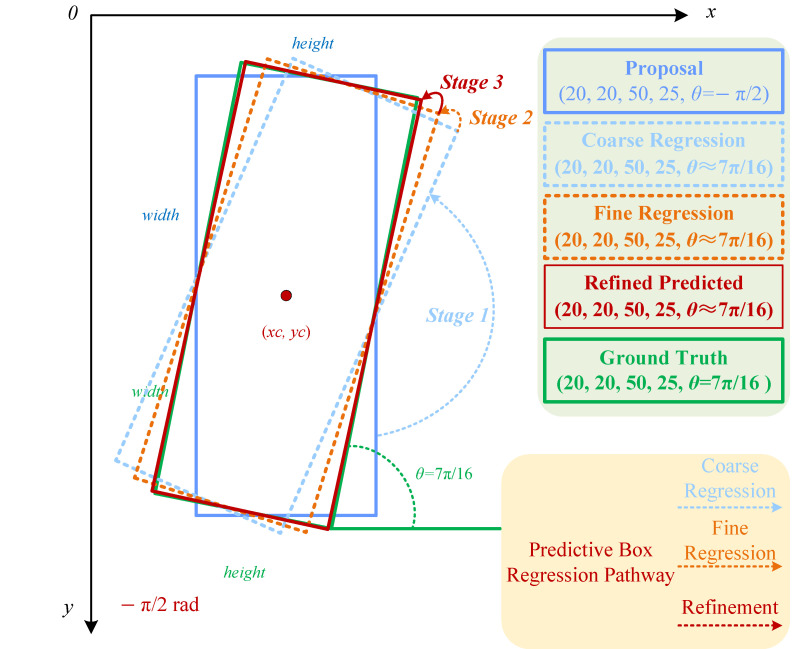
Process of generating predicted boxes by the Spatially Adaptive Angle-aware Network.

Here, F(x,b) represents the regression equation, *x* denotes the image features corresponding to the candidate bounding boxes, and $f_{1},f_{2},f_{3}$ represent the regression functions for the three stages. It is important to note that each regression function $f_{t}$ (t=1,2,3) in the cascade is specifically trained for the optimization of the corresponding bounding box $b^{t}$ (t=1,2,3) at its respective stage. This approach gradually improves the generated predicted bounding boxes through coarse regression, fine-tuning, and precision refinement. The entire model learns automatically based on the training sample set $\left\{G_{i},B_{i}\right\}$, requiring no further manual intervention, achieving the goal of adaptive angle regression. The regression process is illustrated in Figure 3. In an ideal scenario, the true target box has an angle of $\theta =\frac{7}{16}\pi$. After being filtered by the RPN module, a proposal box is generated that overlaps the centroid of the ground truth box, matching its width and height, but with an angle of $\theta =-\frac{1}{2}\pi$. Initially, Stage 1 performs a large-angle transformation, coarsely regressing the proposal box to the light blue Coarse Regression box. Subsequently, Stages 2 and 3 refine the regression further, resulting in the Refined Predicted box that closely aligns with the ground truth box.

**Figure 4:**
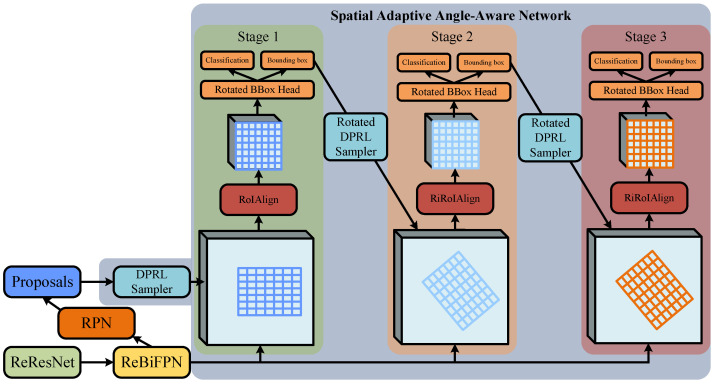
Spatially Adaptive Angle-aware Network structure.

Based on the concept of adaptive angle-regression bounding boxes mentioned above, we have designed the ${\mathrm{SA}}^{3}$ Network, as depicted in Figure 4. The input to the ${\mathrm{SA}}^{3}$ Network is the candidate bounding boxes generated by RPN, and the output consists of the predicted bounding boxes and the classification of those boxes. The processing flow of the ${\mathrm{SA}}^{3}$ Network is as follows: **Stage One: Rotation Bounding Box Regression.** The primary function is to regress horizontal candidate bounding boxes generated by RPN into rotated bounding boxes. The process involves filtering horizontal candidate bounding boxes through the DPRL sampler. Subsequently, RoI Align extracts the feature maps of RoIs. Following the practices of many scholars in the field [11,20,21,23,30], these feature maps are resized to 7×7 (a compromise between computational complexity, feature expressiveness, and simplicity of network design) to facilitate subsequent classification and bounding box regression operations. These fixed-size feature map blocks are fed into the Rotated BBox Head for convolution and fully connected operations, resulting in the regression-generated rotated predicted bounding boxes and their respective classifications; **Stages Two and Three: Rotation Bounding Box Angle Fine-tuning.** The main function is to fine-tune various parameters of the rotated bounding boxes generated in the first stage. The process is similar to the first stage, where rotated bounding boxes are filtered through the Rotated DPRL sampler. Next, the RiRoI Align extracts feature maps corresponding to the rotated candidate bounding boxes. These feature maps are processed through the Rotated BBox Head for convolution and fully connected operations, producing refined rotated predicted bounding boxes and their respective classifications.

#### 2.1.2. Boundary Box Regression Class Strategy Selection

Boundary box regression class strategies include class-agnostic and class-aware strategies. Both have their advantages and disadvantages, requiring consideration of specific application scenarios.

**Figure 5:**
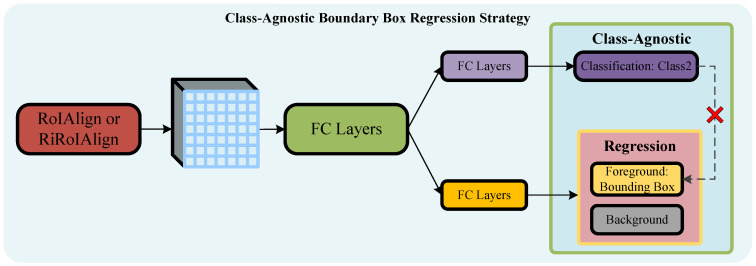
Structure diagram of the class-agnostic strategy regression function in the coarse regression stage.

**Class-Agnostic Boundary Box Regression Strategy.** From a global perspective, in remote sensing images, the orientation of objects of each category is randomly distributed. Given the widely varying aspect ratios of the bounding boxes corresponding to all objects, it is not meaningful to consider the category of objects in the feature map during the coarse regression stage. Therefore, for the regression function $f_{1}\left(x,b\right)$ in the first stage, a class-agnostic strategy is adopted for training the bounding box regression function. The network structure is depicted in Figure 5. The class-agnostic [31] strategy regresses bounding boxes without distinguishing specific categories during the process. This approach enhances recall rates, particularly in cases where there is incomplete annotation in the dataset, improving the model’s robustness. Importantly, it significantly reduces the computational parameter volume of the bounding box regression branch. However, the drawback is that the class-agnostic strategy regresses all objects that the network deems possibly foreground, without determining their specific category. This can lead to multiple detection boxes corresponding to a single complex-patterned object and inaccurate classification. To mitigate the impact on the final detection accuracy, class-aware strategies are employed in the subsequent two stages of regression functions.

Some studies in the general object detection field have demonstrated the effective enhancement of model detection performance using class-agnostic strategies [31]. Experimental findings indicate that, in cases of severe dataset category imbalance, the class-agnostic strategy effectively mitigates the impact of dataset incompleteness on the performance of the object detection model.

**Figure 6:**
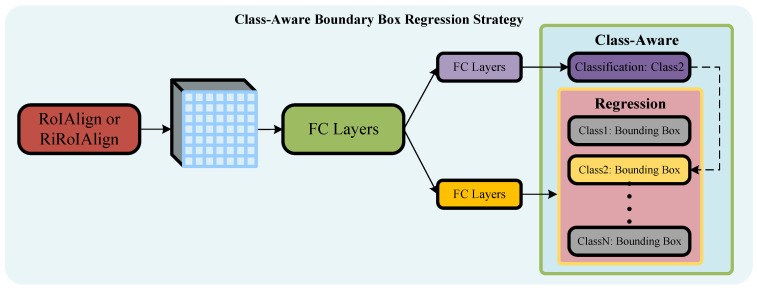
Structure diagram of class-aware strategy regression function in the fine-tuning and precision refinement stages.

**Class-Aware Boundary Box Regression Strategy.** Unlike images captured from a horizontal perspective, objects of different categories in remote sensing images exhibit significant differences in the aspect ratios of their true target boxes. For instance, the aspect ratio of bridges and docks may exceed 1:10, while objects like planes and helicopters may have a ratio of 1:1. Directly applying the research experience of general object detection without considering the actual situation of optical remote sensing images may not be wise. Therefore, in the design of the regression functions $f_{2},f_{3}\left(x,b\right)$ for the fine-tuning and precision refinement stages, a class-aware strategy is employed. The network structure is shown in Figure 6.

When using a class-aware strategy to regress bounding boxes, the regression network individually infers each category by traversing all categories, computing bounding boxes for each category in the feature map block, and outputting the coordinates of bounding boxes for all categories. Then, based on the output results of the classification network branch, the bounding boxes corresponding to the category of the classification network branch are indexed, labeled as detection boxes, and other irrelevant category bounding boxes are discarded, obtaining the final detection results. The advantage of this approach is that it allows the model to undergo fine training for each category, serving as the basis for fine regression and refinement learning, ultimately enabling the model to generate accurate detection boxes for each category’s object. The drawback is that for a dataset with annotated N categories of objects, the computational complexity of the regression network branch is N times that of the class-agnostic strategy, making it less efficient. However, for this project, improving detection accuracy through algorithm design takes precedence, while also considering a reduction in computational complexity. As per the empirical knowledge of generative artificial intelligence, the size of the computed parameter quantity is positively correlated with the actual performance of the model. In experiments, it was observed that although the class-aware strategy incurs a higher computational cost, it does not necessarily achieve better detection performance when the number of categories in the dataset is imbalanced or the total number of targets is small.

In summary, when using the ${\mathrm{SA}}^{3}$ Network, one should consider whether the number of targets in each category in the dataset is balanced, the total number of targets, and other practical issues. A comprehensive judgment should be made regarding whether to adopt a class-aware or class-agnostic strategy during the fine regression and refinement stages of the ${\mathrm{SA}}^{3}$ Network. Inappropriate strategy selection may result in a significant reduction in the model’s detection performance.

### 2.2. Edge-Aware Skewed Bounding Box Loss

A persistent issue in the design of regression losses for rotated object detection is the inconsistency between model training metrics and final evaluation metrics. This inconsistency acts as a bottleneck, necessitating the design of a loss function specifically tailored for rotated object detection. Such a loss function must consider the unique characteristics of this task, especially in terms of the Intersection over Union (IoU), to enhance the model’s sensitivity to changes in angles.

To address this challenge, we introduce a novel regression loss, named the EAS loss. This loss function takes into account the IoU value between the predicted bounding box and the ground truth. The EAS loss effectively mitigates the inconsistency between training and evaluation metrics, enabling the model to accurately regress angles, particularly when the predicted box is close to the ground truth.

#### 2.2.1. EAS Loss Design

To enhance the sensitivity to small angle deviations and improve metric consistency, a natural idea is to use IoU when regressing the angle θ. In this case, the loss calculation is defined as in Equation (2):

(2) $${\mathrm{Loss}}_{IoU}=-\log\left(\mathrm{eps}+\mathrm{IoU}\right),$$

**Figure 7:**
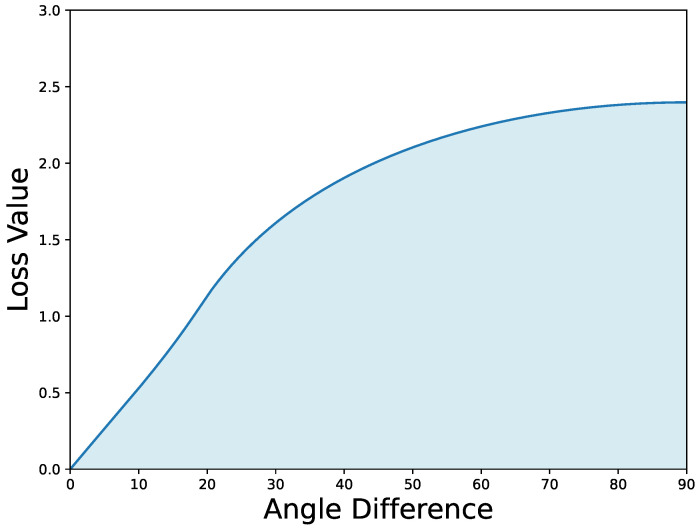
Curve depicting the relationship between IoU loss and angle difference.

However, this introduces a new problem: when the centroid of the predicted box is far from the centroid of the ground truth box, the IoU value will be very small, leading to slow angle regression by the model. Assuming the aspect ratio of the box is 1:6, as shown in Figure 7, even when the centroid of the predicted box and the ground truth box coincide, the slope of the ${\mathrm{Loss}}_{IoU}$ value becomes flat when the angle difference θ exceeds 60°. This reduces the regression efficiency of the model for larger angle differences.

To address this, we introduce an IoU factor into the angle regression loss $L_{\theta }^{*}$ calculation, as shown in Equation (3):

(3) $$L_{\theta }=L_{\theta }^{*}-\log\left(\mathrm{eps}+\mathrm{IoU}\right),$$

Here, $L_{\theta }^{*}$ adopts the calculation method of the Smooth L1 loss, leading to the ideal EAS loss calculation Equation (4):

(4) $$\begin{array}{c} {\mathrm{Loss}}_{\mathrm{EAS}}\left(d_{\theta },t_{\theta },\beta ,\gamma \right)=\left\{\begin{array}{cc} 0.5\cdot \left(\frac{|d_{\theta }-t_{\theta }{|}^{2}}{\beta }-\gamma \cdot \log\left(\mathrm{eps}+\mathrm{IoU}\right)\right), & \mathrm{if}\;|d_{\theta }-t_{\theta }|<\beta \\ |d_{\theta }-t_{\theta }|-0.5\cdot \beta -\gamma \cdot \log\left(\mathrm{eps}+\mathrm{IoU}\right), & \mathrm{otherwise} \end{array}\right. \end{array},$$

**Figure 8:**
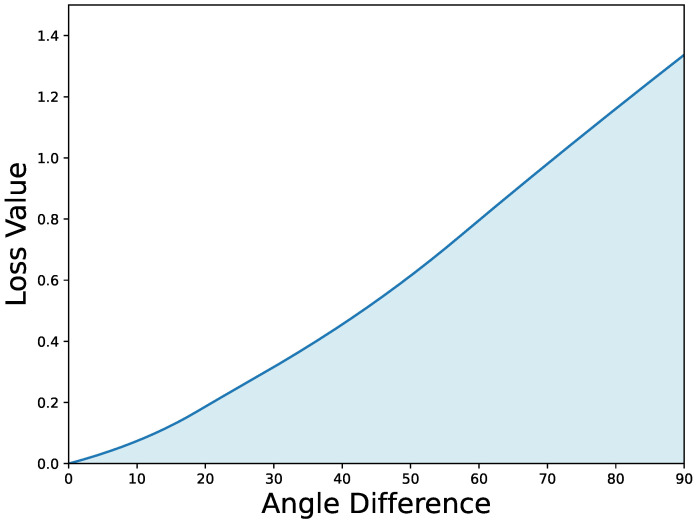
Curve depicting the relationship between EAS loss and angle.

Here, $\mathrm{eps}=10^{-6}$, and β and γ are adjustable variables. When β=1 and γ=1/9, the relationship between ${\mathrm{Loss}}_{\mathrm{EAS}}$ and the angle difference θ is depicted in Figure 8.

When the centroids of the predicted box and the ground truth box do not coincide, the EAS loss, incorporating the IoU factor in the angle regression loss component, prevents ineffective rotations of the predicted box. When the centroids of the predicted box and the ground truth box do coincide, the IoU factor allows the model to quickly and accurately learn to generate more precise predicted boxes as the predicted and ground truth angles approach each other. This makes it an ideal angle regression loss function.

The next issue is how to calculate the IoU value. Here, the IoU between the rotated bounding boxes is termed SkewIoU to distinguish it from horizontal IoU. The definition of SkewIoU is the same as IoU, as shown in Equation (5):

(5) $$\mathrm{SkewIoU}\left(P,G\right)=\frac{\mathrm{Area}(P\cap G)}{\mathrm{Area}(P\cup G)},$$

where *P* and *G* are two rotated bounding boxes. To compute their intersection and union areas accurately, skewed bounding boxes are treated as polygons, and the convex polygon intersection calculation method [5] is employed to precisely calculate SkewIoU. This process involves sorting and combining the coordinates of the vertices and intersection points of the rotated boxes, followed by the application of the triangulation method to calculate the area of the intersection region. The overall computation is relatively complex, with sorting and triangulation being the primary time-consuming factors. In practical applications, libraries such as OpenCV and Shapely are often employed to calculate SkewIoU. The specific implementation and optimizations in these libraries can impact performance, but the fundamental principles of computational complexity remain unchanged.

In general object detection, the IoU loss has long been a focus for effectively mitigating the inconsistency between evaluation metrics (dominated by IoU) and regression loss calculation methods. However, in rotated object detection, due to the computationally expensive nature of SkewIoU calculation, it is primarily used for validation and evaluation and has not been widely adopted in loss functions.

#### 2.2.2. Gaussian Transformation for Approximate IoU Calculation

Recent studies, such as PIoU [32], projection IoU [33], GWD [34], and KLD [35], have explored methods to simulate the approximate SkewIoU loss. Among these, GWD and KLD introduced a Gaussian modeling approach, simplifying the complex SkewIoU calculation into a more efficient process. However, their methods involve nonlinear transformations and hyperparameters in the final loss function design using Gaussian distribution distance metrics, making them not fundamentally SkewIoU. KFIoU [36] is a loss function based on the approximation of SkewIoU and center-point distance, but its performance in the proposed model in this paper is not ideal. As shown in Table 1, after incorporating the KFIoU loss, the model’s AP50, AP75, and mAP all experienced varying degrees of decline. In the original paper, KFIoU achieved satisfactory results with backbone networks like ResNet-152 with large parameter sizes. In contrast, the model in this paper utilizes the ReResNet-50 backbone network with relatively fewer computational parameters, which might be one of the reasons for the performance degradation.

Despite KFIoU’s poor performance in ReBiDet, a simpler and more efficient method for approximating SkewIoU is worth considering. This method converts two rotated bounding boxes into Gaussian distributions, representing the overlapping region with a simpler mathematical formula and calculating the area of the minimum enclosing rectangle of this region. This approach significantly simplifies the original geometric computation while maintaining a high approximation to the exact IoU value [36]. This method adheres to the SkewIoU calculation process, is mathematically rigorous, and does not introduce additional hyperparameters. Although the accuracy of the SkewIoU values obtained is relatively low, precise SkewIoU calculation is not strictly necessary in loss functions; minor errors are tolerable as long as the trend of the approximated SkewIoU matches the true value. Below are the basic steps and derivations for approximating SkewIoU using the Gaussian transformation method [36]:

**Transforming Rotated Rectangular Boxes into Gaussian Distributions.** To begin, the rotated rectangular boxes are transformed into Gaussian distributions G(μ,Σ). Each rectangular box is represented by two parameters: the covariance matrix Σ and the center coordinates μ. For a rotated rectangular box B(x,y,w,h,θ), $\mu =\left[\begin{array}{c} x\\ y \end{array}\right]$, and the covariance matrix is calculated as shown in Equation (6):

(6) $$\Sigma =R\Lambda R^{T},$$

where $R=\left[\begin{array}{cc} \cos\theta & -\sin\theta \\ \sin\theta & \cos\theta \end{array}\right],\;\Lambda =\frac{1}{4}{\left[\begin{array}{cc} w^{2} & 0\\ 0 & h^{2} \end{array}\right]}^{2}$. The final form of the covariance matrix Σ is given in Equation (7):

(7) $$\Sigma =\left[\begin{array}{cc} \frac{w^{2}}{4}\cos^{2}\theta +\frac{h^{2}}{4}\sin^{2}\theta & \frac{\left(w^{2}-h^{2}\right)}{4}\cos\theta \sin\theta \;\\ \frac{\left(w^{2}-h^{2}\right)}{4}\cos\theta \sin\theta & \frac{w^{2}}{4}\sin^{2}\theta +\frac{h^{2}}{4}\cos^{2}\theta \end{array}\right],$$

**Figure 9:**
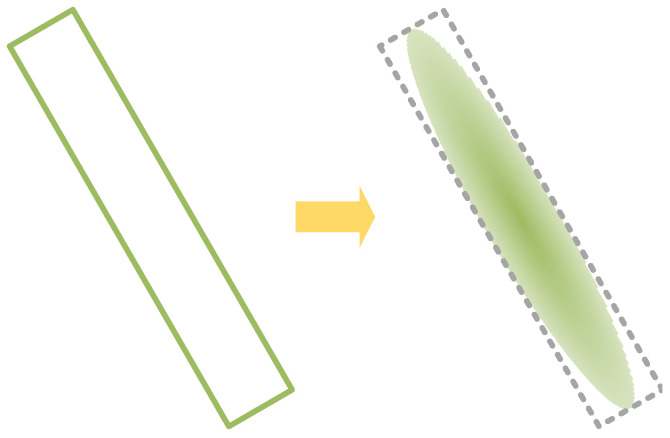
Transformed Gaussian distribution of rotated rectangular boxes.

The transformed Gaussian distribution is illustrated in Figure 9.

**Calculating Overlapping Area of Two Gaussian Distributions.** The overlapping area of two Gaussian distributions is computed by multiplying the Gaussian distributions of the predicted box *P* and the true target box *G* using the Kalman filter’s multiplication rule. Specifically, the predicted box’s Gaussian distribution $G_{P}\left(\mu_{P},\Sigma_{P}\right)$ is treated as the predicted value, and the true target box’s Gaussian distribution $G_{G}\left(\mu_{G},\Sigma_{G}\right)$ is treated as the observed value. This yields the approximate Gaussian distribution $G_{I}\left(\mu_{I},\Sigma_{I}\right)$ for the overlapping region *I*, as shown in Equation (8):

(8) $$\alpha G_{I}\left(\mu_{I},\Sigma_{I}\right)=G_{P}\left(\mu_{P},\Sigma_{P}\right)\cdot G_{G}\left(\mu_{G},\Sigma_{G}\right),$$

Here, $\alpha G_{I}\left(\mu_{I},\Sigma_{I}\right)$ does not have a probability sum of 1 and is not a standard Gaussian distribution. The coefficient α can be expressed by Equation (9):

(9) $$\alpha =G_{\alpha }\left(\mu_{G},\Sigma_{P}+\Sigma_{G}\right)=\frac{1}{\sqrt{\det(2\pi \left(\Sigma_{P}+\Sigma_{G}\right))}}e^{-\frac{1}{2}{\left(\mu_{P}-\mu_{G}\right)}^{T}{\left(\Sigma_{P}+\Sigma_{G}\right)}^{-1}\left(\mu_{P}-\mu_{G}\right)},$$

**Figure 10:**
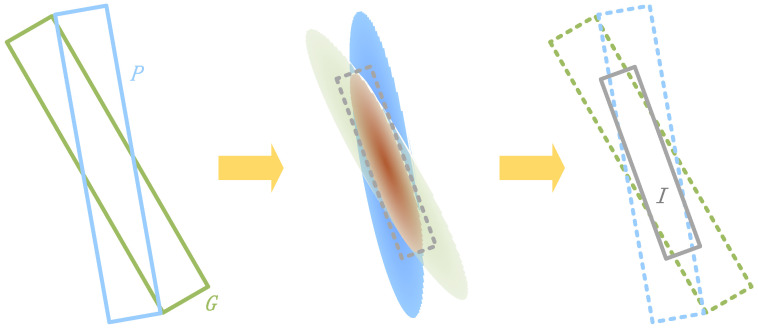
The overlapping area *I* of two Gaussian distributions.

When $\mu_{P}-\mu_{G}\approx 0$, α can be approximated as a constant. Since the EAS loss is computed separately for the centroids of the bounding boxes, we can consider the scenario where the centroids of the predicted box *P* and the ground truth box *G* coincide, i.e., $\mu_{P}=\mu_{G}$. In this case, the parameters $\mu_{I}$ and $\Sigma_{I}$ of the Gaussian distribution $G_{I}\left(\mu_{I},\Sigma_{I}\right)$ for the overlap region *I* are calculated using Equation (10):

(10) $$\mu_{I}=\mu_{P}+K\left(\mu_{G}-\mu_{P}\right),\;\Sigma_{I}=\Sigma_{P}-K\Sigma_{P},$$

where $K=\Sigma_{P}{\left(\Sigma_{P}+\Sigma_{G}\right)}^{-1}$. When the central points of the predicted box *P* and the true target box *G* are close to overlapping ($\mu_{P}=\mu_{G}$), the center point $\mu_{I}$ of the Gaussian distribution for the overlap region *I* coincides with the central points of the predicted box *P* and the true target box *G*, as shown in Figure 10. The covariance matrix $\Sigma_{I}$ of the overlapping area *I* can be approximated using Equation (11):

(11) $$\Sigma_{I}=\Sigma_{P}\left(1-\frac{\Sigma_{P}}{\Sigma_{P}+\Sigma_{G}}\right)=\frac{\Sigma_{P}\Sigma_{G}}{\Sigma_{P}+\Sigma_{G}},$$

It can be observed that the covariance $\Sigma_{I}$ of the overlapping area *I* is influenced by the covariance $\Sigma_{P}$ and $\Sigma_{G}$ of the Gaussian distributions of the predicted box *P* and the true target box *G*.

**Calculating Areas of Externally Circumscribed Rectangles.** The areas of the minimum enclosing rectangles for the predicted box *P*, the ground truth box *G*, and the overlap region *I* are calculated using their respective Gaussian distributions. Since the dimensions of the predicted box *P* and the ground truth box *G* are known, $S_{P}\left(\Sigma_{P}\right)$ and $S_{G}\left(\Sigma_{G}\right)$ can be directly calculated. The area $S_{I}\left(\Sigma_{I}\right)$ can be conveniently derived from the covariance matrix $\Sigma_{I}$, as shown in Equation (12):

(12) $$S_{I}\left(\Sigma_{I}\right)=2^{2}\sqrt{\prod_{i}\mathrm{eig}\left(\Sigma_{I}\right)}=4\cdot \left|\Sigma_{I}^{1/2}\right|=4\cdot \sqrt{|\Sigma_{I}|},$$

**Calculating the Approximate SkewIoU.** The approximate SkewIoU (${\mathrm{SkewIoU}}_{\mathrm{approx}}$) is calculated as follows:

(13) $${\mathrm{SkewIoU}}_{\mathrm{approx}}=\frac{S_{I}\left(\Sigma_{I}\right)}{S_{P}\left(\Sigma_{P}\right)+S_{G}\left(\Sigma_{G}\right)-S_{I}\left(\Sigma_{I}\right)},$$

However, $S_{I}\left(\Sigma_{I}\right)$ is not the exact area of the overlap of the predicted box *P* and the true target box *G*. Since $\alpha G_{I}\left(\mu_{I},\Sigma_{I}\right)$ is not a standard Gaussian distribution and α is treated as a constant in the calculation, Xue Yang et al. derived that the upper bound of $\cdot \frac{S_{I}\left(\Sigma_{I}\right)}{S_{P}\left(\Sigma_{P}\right)+S_{G}\left(\Sigma_{G}\right)-S_{I}\left(\Sigma_{I}\right)}$ is $\frac{1}{3}$ [36]. Using this upper bound, a linear transformation is applied to expand its value range to [0, 1], resulting in the approximate SkewIoU:

(14) $${\mathrm{SkewIoU}}_{\mathrm{approx}}=3\cdot \frac{S_{I}\left(\Sigma_{I}\right)}{S_{P}\left(\Sigma_{P}\right)+S_{G}\left(\Sigma_{G}\right)-S_{I}\left(\Sigma_{I}\right)},$$

This approximation method for ${\mathrm{SkewIoU}}_{\mathrm{approx}}$ demonstrates high consistency with the true ${\mathrm{SkewIoU}}_{\mathrm{plain}}$ while being an ideal method for practical applications [36]. Despite the complexity of the derivation, the implementation is straightforward in the EAS loss function, where ${\mathrm{SkewIoU}}_{\mathrm{approx}}$ is used as a factor for angle regression without introducing additional hyperparameters and not participating in the backpropagation process.

The EAS loss function is given by:

(15) $${\mathrm{Loss}}_{\mathrm{EAS}}\left(d_{\theta },t_{\theta },\beta ,\gamma \right)=\left\{\begin{array}{cc} 0.5\cdot \left(\frac{|d_{\theta }-t_{\theta }{|}^{2}}{\beta }-\gamma \cdot \log\left(\varepsilon +{\mathrm{SkewIoU}}_{\mathrm{approx}}\right)\right) & \mathrm{if}\;|d_{\theta }-t_{\theta }|<\beta \\ |d_{\theta }-t_{\theta }|-0.5\cdot \beta -\gamma \cdot \log\left(\varepsilon +{\mathrm{SkewIoU}}_{\mathrm{approx}}\right) & \mathrm{otherwise} \end{array}\right.,$$

where $\varepsilon =10^{-6}$, and β and γ are adjustable parameters.

## 3. Experimental Results

### 3.1. Datasets

#### 3.1.1. DOTA-v1.5 Dataset

DOTA [37,38], released by Wuhan University in January 2018, currently comprises three versions: DOTA-v1.0, DOTA-v1.5, and DOTA-v2.0.

DOTA-v1.0 [37] includes 2806 images sourced from various platforms, such as Google Earth, JL-1, and GF-2. This dataset contains 188,282 annotated objects across 15 different categories: Plane (PL), Baseball Diamond (BD), Bridge (BR), Ground Track Field (GTF), Small Vehicle (SV), Large Vehicle (LV), Ship (SH), Tennis Court (TC), Basketball Court (BC), Storage Tank (ST), Soccer-Ball Field (SBF), Roundabout (RA), Harbor (HA), Swimming Pool (SP), and Helicopter (HC). The annotations are quadrilaterals defined by four points $\left\{x_{1},y_{1};x_{2},y_{2};x_{3},y_{3};x_{4},y_{4}\right\}$. However, DOTA-v1.0 misses many small-sized objects (approximately 10 pixels or smaller), leading to incomplete annotations. This limitation may result in inaccurate evaluations of object detection model performance [28].

DOTA-v1.5 [38], built upon DOTA-v1.0, expands the number of annotations to 402,089. It also includes additional annotations for the small objects omitted in DOTA-v1.0 and introduces a new category, Container Crane (CC), making it a more challenging dataset. The dataset presents the following difficulties: (1) the pixel size of images varies significantly, ranging from 800×800 to 4000×4000, complicating training on certain GPUs; (2) a high proportion of objects are small, with 98% of objects being smaller than 300 pixels and 57% smaller than 50 pixels [37], resulting in a substantial scale variation between tiny and large objects, complicating detection.

Due to the limited research published using DOTA-v2.0, cross-performance comparisons are challenging; thus, this paper does not utilize DOTA-v2.0. Instead, DOTA-v1.5 is employed for testing and analyzing the proposed methods, as it addresses the shortcomings of DOTA-v1.0 and remains the most commonly used public object detection dataset in the remote sensing field. It is noteworthy that the creators of the DOTA dataset have not publicly released the annotations for the test set, requiring researchers to upload their detection results for evaluation, thereby restricting comprehensive analysis of experimental results.

#### 3.1.2. DFShip Dataset

The DFShip dataset is a fine-grained optical remote sensing ship dataset released by the Big Data and Decision-Making (National) Laboratory for the 2023 National Big Data and Computational Intelligence Challenge [39]. It comprises 41,495 images, all annotated with ship targets. The training set includes 30,285 images with corresponding target annotation files, totaling 120,605 annotated targets across 133 fine-grained categories. This makes DFShip the most detailed and extensive fine-grained ship dataset currently available, posing a significant challenge to the performance of object detection models. Both the preliminary and final test sets consist of 11,210 images, each presenting varying levels of detection difficulty. The organizers did not provide annotation files for the test sets; participants must test locally and upload the packaged test results to a designated server for validation. Since the organizers did not provide a specific name for this dataset, we refer to it as DFShip in this paper.

### 3.2. Setup

The experimental setup closely follows the approach described in the ReBiDet paper, with several key upgrades. The GPU configuration has been enhanced from 2 NVIDIA GTX 3090 Ti to 2 NVIDIA GTX 4090 GPUs. Additionally, the software stack has been updated: CUDA has been upgraded from version 11.8 to 12.0, PyTorch from version 1.11.0 to 1.13.1, torchvision from version 0.12.0 to 0.14.1, and the MMRotate framework from version 0.3.2 to 0.3.4. These updates ensure improved compatibility and facilitate the reproducibility of experiments across different environments.

During training, data augmentation techniques, such as horizontal and vertical flips, were applied. The batch size per GPU was set to 2, resulting in a total batch size of 4. The network was optimized using the Stochastic Gradient Descent (SGD) algorithm with a momentum of 0.9 and a weight decay of 0.0001. For the Region Proposal Network (RPN), the IoU threshold for positive samples was set to 0.7. The horizontal box Non-Maximum Suppression (NMS) threshold was set to 0.7, while the rotation box NMS threshold was set to 0.1.

The DOTA-v1.5 dataset consists of a total of 2806 images, with 1411 images in the training set, 458 images in the validation set, and 937 images in the test set. To ensure fairness in comparison experiments, we followed the practices of many scholars in the field and processed the data accordingly. Since the images in the dataset have varying sizes, the original images from the DOTA-v1.5 dataset were cropped to a size of 1024 × 1024 pixels with a stride of 824 pixels. After cropping, the DOTA-v1.5 dataset contains 15,749 images in the training set, 5297 images in the validation set, and 10,833 images in the test set. Both the training and validation sets were used for model training. Additionally, we performed multiple scale augmentation on the dataset, resizing the original 2806 images to three scales: 0.5, 1.0, and 1.5. These resized images were then cropped to 1024 × 1024 pixels with a stride of 524 pixels, resulting in a final dataset of 416,651 images for training and 71,888 images for testing. The model was trained for 12 epochs, with an initial learning rate of 0.01, and it was divided by 10 at epochs 9 and 11.

The DOTA-v1.5 dataset consists of a total of 2806 images, with 1411 images in the training set, 458 images in the validation set, and 937 images in the test set. To ensure fairness in comparative experiments, we followed established practices and processed the data accordingly. Given the varying sizes of images in the dataset, the original images were cropped to a size of 1024 × 1024 pixels with a stride of 824 pixels. After cropping, the DOTA-v1.5 dataset was expanded to include 15,749 images in the training set, 5297 images in the validation set, and 10,833 images in the test set. Both the training and validation sets were utilized for model training. Additionally, multiple scale augmentations were performed, resizing the original 2806 images to three scales: 0.5, 1.0, and 1.5. These resized images were cropped to 1024 × 1024 pixels with a stride of 524 pixels, resulting in a final dataset of 416,651 images for training and 71,888 images for testing. The model was trained for 12 epochs, with an initial learning rate of 0.01, which was reduced by a factor of 10 at epochs 9 and 11.

For the DFShip dataset, the original images are typically 1024 × 1024 in size and do not require additional cropping. Since the competition organizer’s validation server only provides mAP at an IoU threshold of 0.5, the original training set was randomly split in a 4:1 ratio for this study. This resulted in a training set with 24,228 images and 96,644 annotated targets, and a test set with 6057 images and 23,961 annotated targets. The model was trained for 36 epochs, with an initial learning rate of 0.01, which was reduced by a factor of 10 at epochs 24 and 33.

### 3.3. Results and Analysis

#### 3.3.1. Ablation Experiments of ${\mathrm{SA}}^{3}$ Network

To evaluate the effectiveness of the proposed ${\mathrm{SA}}^{3}$ Network, ablation experiments were conducted using ReDet and ReBiDet as baseline models. These experiments aimed to assess the impact of incorporating class-aware and class-agnostic bounding box regression strategies.

In these ablation experiments, the ${\mathrm{SA}}^{3}$ Network was integrated into both ReDet and ReBiDet models to objectively evaluate its contribution. Due to the absence of ground truth annotations for the test set of the DOTA dataset, the trained models’ results were packaged and uploaded. The detection metrics were then verified by the DOTA-v1.5 dataset authors using their designated servers.

Table 2 presents a performance comparison of the ReDet and ReBiDet models with and without the ${\mathrm{SA}}^{3}$ Network. The integration of the ${\mathrm{SA}}^{3}$ Network results in improved detection performance for both models. The mean average precision (mAP) reported in the table is computed using the COCO evaluation method, which averages the average precision (AP) values across all possible IoU thresholds. AP50 and AP75 refer to the AP at IoU thresholds of 0.5 and 0.75, respectively.

The results indicate that integrating the ${\mathrm{SA}}^{3}$ Network leads to improvements in mAP, AP50, and AP75 for both the ReDet and ReBiDet models. For ReDet, the class-aware strategy increases AP50 by 0.92% and AP75 by 0.84%. The class-agnostic strategy, however, shows even more significant improvements, with AP50 increasing by 1.28% and AP75 by 2.38%. Similarly, for ReBiDet, the class-aware strategy results in AP50 increasing by 0.68% and AP75 by 0.4%, while the class-agnostic strategy yields an increase of 1.33% in AP50 and 1.59% in AP75.

These results demonstrate that the ${\mathrm{SA}}^{3}$ Network significantly enhances detection accuracy, particularly at higher IoU thresholds. This improvement underscores the network’s ability to optimize model precision for predicting rotated bounding boxes.

**Figure 11:**
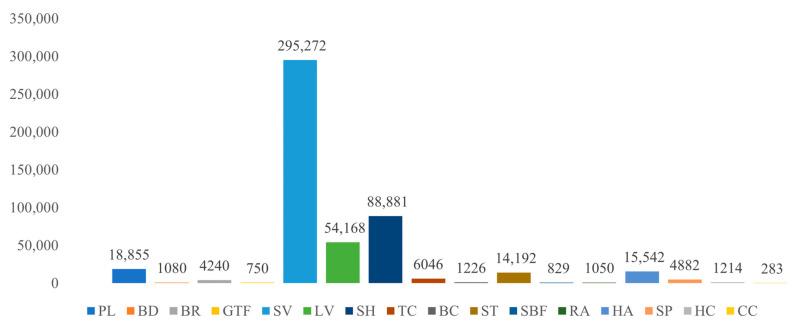
Distribution of various object classes in the DOTA-v1.5 training and validation sets.

Despite the expectation that the class-aware strategy would provide more accurate results due to its tailored approach for each object class, the experimental outcomes show the opposite. The class-agnostic strategy performs better, which can be attributed to the imbalanced class distribution in the DOTA-v1.5 dataset. Some classes, such as Container Crane (CC), have very few instances compared to others like Small Vehicle (SV), which has a large number of instances. Figure 11 illustrates this imbalance, with CC having only 283 instances and SV having 295,272 instances. In such cases, the class-agnostic strategy is more suitable.

**Figure 12:**
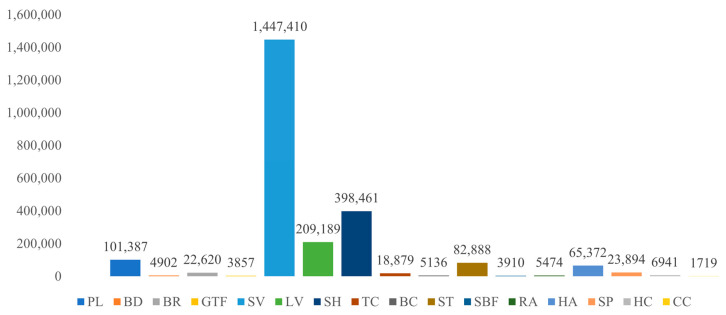
Distribution of various object classes in the augmented DOTA-v1.5 training and validation sets.

To further investigate the effectiveness of the ${\mathrm{SA}}^{3}$ Network and the rationale behind strategy selection, we performed random rotation and offline multi-scale augmentations on the DOTA-v1.5 dataset. The class distribution after augmentation, shown in Figure 12, remains uneven but with an increased minimum instance count for Container Crane (CC), which rises to 1719. This theoretically enhances training effectiveness.

Table 3 displays the ablation experiment results using the augmented DOTA-v1.5 dataset. With the ${\mathrm{SA}}^{3}$ Network, the class-aware strategy shows significant improvements over the ReDet baseline, with AP50 increasing by 1.00% and AP75 by 1.67%. The class-agnostic strategy results in AP50 increasing by 0.68% and AP75 by 1.64%. For ReBiDet, the class-aware ${\mathrm{SA}}^{3}$ Network leads to an increase of 0.54% in AP50 and 0.85% in AP75, contrasting with the previous results where the class-agnostic strategy showed higher effectiveness.

The augmented dataset, despite its imbalances, now provides sufficient instances for each class, making the class-aware strategy more effective. Therefore, while the class-agnostic strategy of the ${\mathrm{SA}}^{3}$ Network is more suitable for datasets with significant class imbalances, the class-aware strategy proves advantageous for datasets with adequate instances for each class, even if imbalances exist.

#### 3.3.2. Ablation Experiments of EAS Loss

Initially, we performed ablation experiments using ReDet as the baseline model to assess the effectiveness of the EAS loss function. Table 4 compares the performance of the ReDet and ReDet + ${\mathrm{SA}}^{3}$ models, both with and without the EAS loss. This preliminary verification indicates that the proposed EAS loss function improves the model’s detection performance. The evaluation metrics are defined as previously described. The results reveal that both baseline models, ReDet and ReDet + ${\mathrm{SA}}^{3}$, exhibit varying degrees of improvement in mAP, AP50, and AP75 with the adoption of the EAS loss. Specifically, ReDet shows a 0.60% increase in AP50 and a 0.18% increase in AP75. For ReDet + ${\mathrm{SA}}^{3}$, the class-aware strategy significantly enhances performance, with AP50 increasing by 1.10% and AP75 by 1.60%.

Further ablation experiments were conducted using ReBiDet as the baseline model to corroborate the effectiveness of the EAS loss function. Table 5 presents the performance comparison of the ReBiDet + ${\mathrm{SA}}^{3}$ models with and without EAS loss. Similar to the ReDet results, the ReBiDet + ${\mathrm{SA}}^{3}$ model exhibits notable improvements under the class-aware strategy when utilizing EAS loss: AP50 increases by 1.11%, AP75 by 1.38%, and mAP by 1.63%. The class-agnostic strategy shows smaller improvements, with AP75 and mAP increasing by 0.80% and 0.35%, respectively, while AP50 decreases by 0.62%, consistent with the results obtained with the ReDet model. This observed phenomenon is not coincidental. The EAS loss function facilitates more comprehensive training of the model’s bounding box regression branch, whereas the class-agnostic strategy treats all categories as a single class, which can be less effective when training samples are insufficient. This fundamental conflict between the mechanisms explains the reduced generalization capability of the ${\mathrm{SA}}^{3}$ Network under the class-agnostic strategy when influenced by EAS loss.

These experimental results indicate that the EAS loss function enhances the training effectiveness of the model’s bounding box regression branch and optimizes the ${\mathrm{SA}}^{3}$ Network, particularly improving the learning efficiency of the class-aware strategy. This ensures that the model can be effectively trained even with imbalanced categories and fewer samples.

We then evaluated the performance of the EAS loss function on a dataset with a larger number of training samples by applying random rotation and offline multi-scale augmentation to the DOTA-v1.5 dataset.

Initially, ablation experiments were conducted using ReDet + ${\mathrm{SA}}^{3}$ as the baseline model. Given that the class-agnostic strategy performs relatively poorly on the unaugmented DOTA-v1.5 dataset, we employed the class-aware strategy for ${\mathrm{SA}}^{3}$ in this case. Table 6 compares the performance of the ReDet + ${\mathrm{SA}}^{3}$ models with and without EAS loss. The results show slight improvements across all performance indicators: AP50 increased by 0.25%, AP75 by 0.16%, and mAP by 0.36%. These improvements are significantly less than those observed with the unaugmented DOTA-v1.5 dataset.

Similarly, ablation experiments using ReBiDet + ${\mathrm{SA}}^{3}$ as the baseline model were conducted, maintaining the class-aware strategy for ${\mathrm{SA}}^{3}$ as explained above. Table 7 presents a performance comparison of the ReBiDet + ${\mathrm{SA}}^{3}$ models with and without EAS loss. As with the ReDet + ${\mathrm{SA}}^{3}$ model, the EAS loss yields minor improvements: AP50 increased by 0.35%, AP75 decreased by 0.12%, and mAP increased by 0.55%. These improvements are again notably smaller compared to those seen with the unaugmented DOTA-v1.5 dataset.

The diminished advantage of the EAS loss function with an increased number of effective training samples is consistent with our previous observations. This effect is evident in both the ReDet + ${\mathrm{SA}}^{3}$ and ReBiDet + ${\mathrm{SA}}^{3}$ models, with the latter exhibiting stronger feature extraction capabilities. The results suggest that enhanced feature extraction and sample selection strategies cannot maintain the significant advantage of EAS loss. Nevertheless, the performance of both ReDet/ReBiDet + ${\mathrm{SA}}^{3}$ models still benefits from the use of EAS loss.

In summary, the EAS loss function effectively improves the learning efficiency of the model’s regression branch for bounding box angle regression. It ensures adequate training even with imbalanced categories and fewer samples. Specifically, the class-aware strategy of the ${\mathrm{SA}}^{3}$ Network benefits significantly from the EAS loss, enhancing the performance metrics, such as AP50, AP75, and mAP. In scenarios with insufficient training samples for certain categories, the ${\mathrm{SA}}^{3}$ Network does not need to choose between class-aware and class-agnostic strategies. As the number of effective training samples increases and each category has sufficient instances, the advantage of the EAS loss diminishes but remains relative to the Smooth L1 loss. These results validate the effectiveness of the EAS loss function in various scenarios.

#### 3.3.3. Multi-IoU Threshold Comparison Experiment on the DFShip Dataset

The DOTA series datasets, depending on the version, include 15 or 16 categories, such as airplanes and cars, with ships being one of the categories. To further demonstrate the effectiveness of the proposed solutions, design concepts, and research methods in addressing ship detection issues, we conducted validation experiments on the DFShip dataset, which specializes in ship detection. This subsection provides a horizontal comparison of the ReBiDet + ${\mathrm{SA}}^{3}$ + EAS model proposed in this paper with other state-of-the-art models on the DFShip dataset. We selected and reproduced several leading models from the DOTA series datasets for this comparison, including CSL [26], R3Det [12], S2A-Net [25], Oriented R-CNN [22], and our baseline model ReDet [23]. These models are among the most advanced in the field. Due to the unavailability of annotated test data from the official source, we split the original training set into training and test sets in a 4:1 ratio for the experiments, focusing on the precision of generated bounding boxes, specifically mAP at high IoU thresholds.

Table 8 presents the experimental results. At low threshold conditions, such as IoU = 0.5, the performance advantage of our proposed model over other advanced models is not significant. This is partly due to the dataset characteristics, which include clear images, accurate annotations, and a large number of training samples, all of which generally enhance the detection performance of the models. However, under high threshold conditions, such as IoU = 0.75, our model demonstrates a clear advantage with a detection accuracy of 98.28%, outperforming all other models in the comparison and surpassing the baseline model ReDet by 2.22%. The detection accuracy at IoU = 0.75 is only 0.56% lower than at IoU = 0.5.

**Figure 13:**
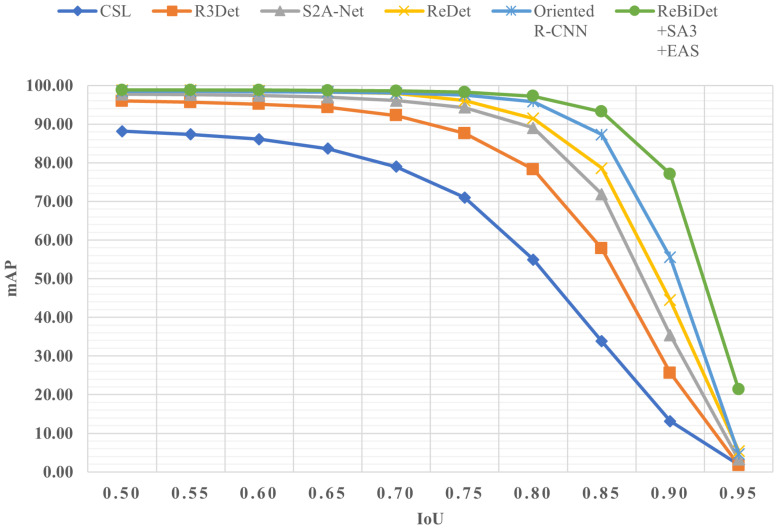
Horizontal comparison of model performance on the DFShip dataset at IoU thresholds from 0.5 to 0.95. The mAP is calculated using the all-point interpolation method.

At an even higher threshold of IoU = 0.85, the detection accuracy of ReBiDet + ${\mathrm{SA}}^{3}$ + EAS remains robust at 93.18%, while the performance of all other models significantly declines. At IoU = 0.95, ReBiDet + ${\mathrm{SA}}^{3}$ + EAS maintains a detection accuracy of 19.18%, whereas the other models’ accuracies drop to single digits. Figure 13 visually presents the comparison results from Table 8. These results confirm that our model not only achieves high detection accuracy but also generates more precise bounding boxes. This validates our research approach and demonstrates the effectiveness of the design methods and strategies employed.

#### 3.3.4. Results

This subsubsection presents a comprehensive comparison of the ReDet and ReBiDet models, enhanced with the ${\mathrm{SA}}^{3}$ Network and EAS loss, against several state-of-the-art models on the DOTA-v1.5 dataset. The objective is to evaluate the effectiveness of the proposed methods by performing a horizontal comparison with other advanced models published in recent years. All comparison models are sourced from reputable journals and conferences, and their performance metrics are cited directly.

Table 9 provides a detailed comparison of the ReDet and ReBiDet models integrated with the ${\mathrm{SA}}^{3}$ Network and EAS loss against other state-of-the-art models on the DOTA-v1.5 dataset. On the unaugmented DOTA-v1.5 dataset, the ReBiDet + ${\mathrm{SA}}^{3}$ + EAS model, using the class-aware strategy, surpasses all compared models with a mean average precision (mAP) of 71.28%. This is slightly higher than the ReBiDet + ${\mathrm{SA}}^{3}$ model utilizing the class-agnostic strategy. Notably, ReBiDet + ${\mathrm{SA}}^{3}$ + EAS ranks within the top three for average precision (AP) values across 15 subcategories and achieves first place in 10 subcategories. It excels in categories with a high aspect ratios, such as bridges (BR) and harbors (HA), as well as in categories with low aspect ratios, such as small vehicles (SV) and large vehicles (LV), which share some characteristics with ships.

When evaluated on the augmented DOTA-v1.5 dataset, which includes random rotation and offline multi-scale enhancements, the ReBiDet + ${\mathrm{SA}}^{3}$ + EAS model demonstrates superior performance, achieving a mAP of 78.85%. It ranks in the top three for accuracy in 10 subcategories, including ships (SH). These results underscore the effectiveness of the proposed approach in significantly enhancing the target detection performance of existing models in optical remote sensing images.

## 4. Discussion

The experimental results confirm that the proposed ${\mathrm{SA}}^{3}$ Network demonstrates excellent precision and localization performance in detecting rotated objects. The EAS loss function, designed to enhance edge perception of inclined bounding boxes, significantly improves the model’s learning efficiency in rotated object detection tasks. This leads to more accurate localization of detection boxes, highlighting the effectiveness of the design approach and methodology presented in this paper.

The ${\mathrm{SA}}^{3}$ Network incorporates a cascaded regression branch. Initially, a coarse regression branch converts horizontal bounding boxes generated by the Region Proposal Network (RPN) into rotated bounding boxes based on the features of the detected objects. Depending on the task scenario, the network employs either a class-aware or class-agnostic strategy. Fine regression and refinement branches further optimize the angle parameters of the rotated bounding boxes, thereby enhancing the fitting accuracy of the final detection boxes.

The inclusion of EAS loss in the angle regression branch introduces an Intersection over Union (IoU) factor, which mitigates the mismatch between traditional loss functions and evaluation metrics. This results in overall improvements across various detection accuracy metrics. The EAS loss adjusts the function gradient of the angle regression branch, addressing the issue of limited learning when the predicted box angle is close to the true target angle. Consequently, the model becomes more sensitive towards the end of the angle regression branch, producing more accurate predicted box angles and significantly improving the AP75 accuracy metric.

**Figure 14:**
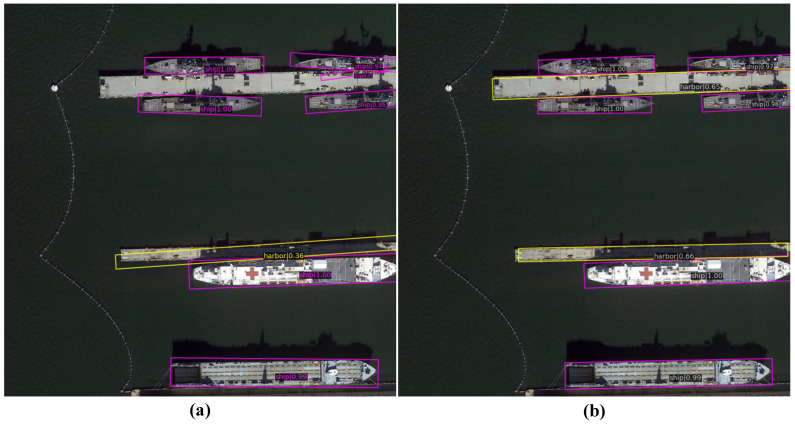
Detection results on an image from the DOTA-v1.5 dataset. (**a**) Detection result of ReBiDet. (**b**) Detection result of the proposed ReBiDet + ${\mathrm{SA}}^{3}$.

Figure 14 illustrates the detection results for the same port remote sensing image. In Figure 14a, the detection results of the ReBiDet model are shown. The ReBiDet model struggles with accurate bounding box localization for elongated objects, such as ships, due to issues with discontinuous boundaries. Figure 14b displays the results after integrating the ${\mathrm{SA}}^{3}$ Network. It is evident that ReBiDet + ${\mathrm{SA}}^{3}$ generates more precise bounding boxes for ships and docks, and improves detection confidence for the ships in the upper-right corner and the dock in the middle-lower part of the image. Additionally, the improved localization accuracy leads to a higher detection rate and a lower false detection rate. After incorporating the ${\mathrm{SA}}^{3}$ Network, the harbor in the middle-upper part of the image is correctly detected, and previously misidentified objects on the harbor are now accurately classified as irrelevant.

However, the proposed method has some limitations concerning computational parameters and inference speed. The ReBiDet + ${\mathrm{SA}}^{3}$ + EAS model increases the number of computational parameters by 13.99 M compared to the baseline ReBiDet model. While the EAS loss itself does not significantly contribute to this increase, the additional parameters primarily result from the ${\mathrm{SA}}^{3}$ Network. This increase in computational parameters leads to an additional 3.5 ms per image on an RTX4090 platform, corresponding to a decrease of 1.8 FPS. Although these computational trade-offs are acceptable in many scenarios given the improvements in detection accuracy, reducing computational parameters and enhancing inference speed while maintaining high detection accuracy remains a critical area for future research.

In conclusion, the methods proposed in this paper are highly effective in improving the detection accuracy of randomly oriented objects, particularly those with elongated contour features, in remote sensing applications. These advancements provide valuable insights for enhancing ship detection capabilities in optical remote sensing images.

## 5. Conclusions

This paper addresses the challenges associated with significant deviations in final detection boxes due to a wide range of angles, as well as the imprecision inherent in traditional angle regression losses. To tackle these issues, we propose the ${\mathrm{SA}}^{3}$ Network and EAS loss. The ${\mathrm{SA}}^{3}$ Network employs a hierarchical regression structure that includes coarse, fine, and refinement stages to progressively optimize the angle parameters of rotated bounding boxes. The EAS loss introduces the SkewIoU factor, calculated using Gaussian transformation, to enhance the precision of angle regression losses. This approach improves both training efficiency and model performance, particularly under high IoU threshold conditions.

Experimental results validate the effectiveness of the ${\mathrm{SA}}^{3}$ Network and EAS loss. The proposed methods significantly improve detection accuracy, especially for rotated objects in optical remote sensing images.

Future work will focus on enhancing the interpretability of rotation-equivariant convolutional neural networks. Understanding the operational mechanisms of these networks presents a significant research opportunity. Our goal is to gain a more intuitive understanding of the features extracted by these networks. We aim to explore techniques for visualizing rotation-equivariant features, which will facilitate a deeper analysis of their limitations and potential improvement strategies.

## Figures and Tables

**Table 1:** Experimental results of the ReBiDet model and KFIoU loss on the DOTA-v1.5 dataset.

Method	mAP	AP50	AP75
ReBiDet [28]	41.15	69.48	42.54
ReBiDet + KFIoU	36.70	68.40	33.62
ReBiDet * [28]	49.26	77.96	52.53
ReBiDet * + KFIoU	46.09	77.06	48.14

* Indicates training and testing on DOTA-v1.5 with random rotation augmentation and offline multi-scale augmentation.

**Table 2:** Experimental results of the ${\mathrm{SA}}^{3}$ Network with two strategies on the DOTA-v1.5 dataset.

Method	mAP	AP50	AP75
ReDet [23]	-	66.86	-
ReDet *	41.00	68.02	42.10
ReDet * + ${\mathrm{SA}}^{3}$ Class-aware	41.23	68.94	42.94
ReDet * + ${\mathrm{SA}}^{3}$ Class-agnostic	41.91	69.30	44.48
ReBiDet [28]	41.15	69.48	42.54
ReBiDet + ${\mathrm{SA}}^{3}$ Class-aware	41.32	70.16	42.94
ReBiDet + ${\mathrm{SA}}^{3}$ Class-agnostic	42.48	71.21	44.13

* Denotes the detection results reproduced on the experimental platform in this paper. Note: The training and inference of models in this table were directly conducted using DOTA-v1.5 for both training and inference, without applying random rotation augmentation and offline multi-scale augmentation to the dataset.

**Table 3:** Experimental results of the ${\mathrm{SA}}^{3}$ Network with two strategies on the augmented DOTA-v1.5 dataset.

Method	mAP	AP50	AP75
ReDet [23]	-	76.80	-
ReDet *	48.13	76.95	50.99
ReDet * + ${\mathrm{SA}}^{3}$ Class-aware	49.38	77.95	52.66
ReDet * + ${\mathrm{SA}}^{3}$ Class-agnostic	49.02	77.63	52.63
ReBiDet [28]	49.26	77.96	52.53
ReBiDet + ${\mathrm{SA}}^{3}$ Class-aware	49.58	78.50	53.38
ReBiDet + ${\mathrm{SA}}^{3}$ Class-agnostic	49.07	77.84	52.11

* Denotes the detection results reproduced on the experimental platform in this paper. Note: The training and inference of models in this table were conducted using DOTA-v1.5, with both random rotation augmentation and offline multi-scale augmentation applied during training and inference.

**Table 4:** EAS loss experiment results based on the ReDet model in the DOTA-v1.5 dataset.

Method	mAP	AP50	AP75
ReDet [23]	-	66.86	-
ReDet *	41.00	68.02	42.10
ReDet * + EAS	41.27	68.62	42.28
ReDet * + ${\mathrm{SA}}^{3}$ Class-aware	41.23	68.94	42.94
ReDet * + ${\mathrm{SA}}^{3}$ Class-aware + EAS	42.34	70.04	44.54
ReDet * + ${\mathrm{SA}}^{3}$ Class-agnostic	41.91	69.30	44.48
ReDet * + ${\mathrm{SA}}^{3}$ Class-agnostic + EAS	42.32	68.91	44.81

* Indicates the detection results reproduced on the experimental platform in this paper. Note: The training and inference of the models in this table were conducted directly using the DOTA-v1.5 dataset, without applying random rotation augmentation or offline multi-scale enhancement. The mAP in the table follows the COCO calculation method, representing the average AP across all possible IoU thresholds. AP50 refers to the AP when the IoU threshold is 0.5, while AP75 denotes the average precision at an IoU threshold of 0.75.

**Table 5:** Experimental results of EAS loss on the ReBiDet model in the DOTA-v1.5 dataset.

Method	mAP	AP50	AP75
ReBiDet + ${\mathrm{SA}}^{3}$ Class-aware	41.32	70.16	42.94
ReBiDet + ${\mathrm{SA}}^{3}$ Class-aware + EAS	42.95	71.28	44.33
ReBiDet + ${\mathrm{SA}}^{3}$ Class-agnostic	42.48	71.21	44.13
ReBiDet + ${\mathrm{SA}}^{3}$ Class-agnostic + EAS	42.83	70.59	44.93

Note: The training and inference of the models in this table were conducted directly using DOTA-v1.5, without applying random rotation augmentation or offline multi-scale enhancement.

**Table 6:** Experimental results of EAS loss on the ReDet model in the augmented DOTA-v1.5 dataset.

Method	mAP	AP50	AP75
ReDet [23]	-	76.80	-
ReDet *	48.13	76.95	50.99
ReDet * + ${\mathrm{SA}}^{3}$	49.38	77.95	52.66
ReDet * + ${\mathrm{SA}}^{3}$ + EAS	49.74	78.20	52.82

* indicates the reproduction of detection results on the experimental platform in this paper. Note: the ${\mathrm{SA}}^{3}$ Networks in this table adopt the class-aware strategy, and the model is trained and tested using random rotation augmentation and offline multi-scale enhancement on the DOTA-v1.5 dataset.

**Table 7:** Experimental results of EAS loss on the ReBiDet model in the augmented DOTA-v1.5 dataset.

Method	mAP	AP50	AP75
ReBiDet [28]	49.26	77.96	52.53
ReBiDet + ${\mathrm{SA}}^{3}$	49.58	78.50	53.38
ReBiDet + ${\mathrm{SA}}^{3}$ + EAS	50.13	78.85	53.26

Note: the ${\mathrm{SA}}^{3}$ Network in this table adopts the class-aware strategy, and the model is trained and tested using random rotation augmentation and offline multi-scale enhancement on the DOTA-v1.5 dataset.

**Table 8:** Horizontal comparison of model performance on the DFShip dataset at IoU thresholds from 0.5 to 0.95.

IoU	CSL	R3Det	S2A-Net	ReDet	Oriented R-CNN	ReBiDet + SA^3^ + EAS
0.50	88.14	95.94	97.72	98.76	98.52	**98.81**
0.55	87.33	95.61	97.59	98.73	98.52	**98.81**
0.60	86.06	95.17	97.38	98.62	98.41	**98.76**
0.65	83.63	94.35	96.96	98.32	98.31	**98.68**
0.70	78.96	92.22	96.07	97.89	98.04	**98.56**
0.75	70.99	87.60	94.27	96.06	97.56	**98.22**
0.80	54.92	78.25	89.01	91.45	95.79	**97.20**
0.85	33.81	57.78	71.88	78.58	87.30	**93.18**
0.90	13.10	25.62	35.37	44.43	55.52	**77.05**
0.95	1.89	1.73	3.18	5.44	4.66	**21.30**
mAP	59.88	72.43	77.94	80.83	83.26	**88.06**

Note: The mAP is calculated using the all-point interpolation method. The red bold font represents the highest detection accuracy value at the same threshold in the horizontal comparison.

**Table 9:** Comparisons with state-of-the-art methods on DOTA-v1.5 OBB task.

Method	PL	BD	BR	GTF	SV	LV	SH	TC	BC	ST	SBF	RA	HA	SP	HC	CC	mAP
single-scale:																	
RetinaNet-O [40]	71.43	77.64	42.12	64.65	44.53	56.79	73.31	90.84	76.02	59.96	46.95	69.24	59.65	64.52	48.06	0.83	59.16
FR-O [37]	71.89	74.47	44.45	59.87	51.28	68.98	79.37	90.78	77.38	67.50	47.75	69.72	61.22	65.28	60.47	1.54	62.00
Mask R-CNN [30]	76.84	73.51	49.90	57.80	51.31	71.34	79.75	90.46	74.21	66.07	46.21	70.61	63.07	64.46	57.81	9.42	62.67
HTC [29]	77.80	73.67	51.40	63.99	51.54	73.31	80.31	90.48	75.12	67.34	48.51	70.63	64.84	64.48	55.87	5.15	63.40
CMR [29]	77.77	74.62	51.09	63.44	51.64	72.90	79.99	90.35	74.90	67.58	49.54	72.85	64.19	64.88	55.87	3.02	63.41
DAFNe [41]	-	-	-	-	-	-	-	-	-	-	-	-	-	-	-	-	64.76
FR OBB [37] + RT [11]	71.92	76.07	51.87	69.24	52.05	75.18	80.72	90.53	78.58	68.26	49.18	71.74	67.51	65.53	62.16	9.99	65.03
ReDet [23]	79.20	82.81	51.92	71.41	52.38	75.73	80.92	90.83	75.81	68.64	49.29	72.03	73.36	**70.55**	63.33	**11.53**	66.86
ReDet + ${\mathrm{SA}}^{3}$	80.12	83.54	**54.08**	72.56	**52.76**	77.18	87.63	**90.87**	84.05	**74.25**	62.07	**73.77**	75.71	65.61	**67.31**	7.26	69.30
ReBiDet [28]	**80.54**	82.90	53.62	**74.55**	52.55	**79.65**	87.53	90.84	**84.57**	72.93	**65.02**	73.05	**75.87**	65.56	65.18	7.32	69.48
ReDet + ${\mathrm{SA}}^{3}$ + EAS	**80.21**	**84.25**	53.50	72.53	52.74	77.04	**87.81**	**90.88**	83.91	69.34	64.26	**73.33**	75.84	66.24	64.80	9.09	**70.04**
ReBiDet + ${\mathrm{SA}}^{3}$	**80.70**	**83.67**	**54.89**	**74.58**	**57.99**	**79.93**	**88.38**	**90.87**	**85.03**	**74.26**	**66.06**	73.15	**76.79**	**70.09**	**67.32**	**15.70**	**71.21**
ReBiDet + ${\mathrm{SA}}^{3}$+EAS	80.12	**83.79**	**56.21**	**73.10**	**58.27**	**80.52**	**88.09**	**90.89**	**84.12**	**74.00**	**67.20**	**75.04**	**77.04**	**70.72**	**67.98**	**13.33**	**71.28**
multi-scale:																	
DAFNe [41]	-	-	-	-	-	-	-	-	-	-	-	-	-	-	-	-	71.99
OWSR * [42]	-	-	-	-	-	-	-	-	-	-	-	-	-	-	-	-	74.90
RTMDet-R-tiny * [43]	88.14	83.09	51.80	77.54	65.99	82.22	89.81	**90.88**	80.54	81.34	64.64	71.51	77.13	**76.32**	72.11	46.67	74.98
RTMDet-R-s * [43]	88.14	85.82	52.90	82.09	65.58	81.83	89.78	90.82	83.31	82.47	68.51	70.93	78.00	75.77	73.09	47.32	76.02
RTMDet-R-m * [43]	**89.07**	**86.71**	52.57	**82.47**	66.13	82.55	89.77	**90.88**	84.39	**83.34**	69.51	73.03	77.82	**75.98**	**80.21**	42.00	76.65
ReDet * [23]	88.51	**86.45**	**61.23**	81.20	67.60	83.65	**90.00**	90.86	84.30	75.33	71.49	72.06	78.32	74.73	76.10	46.98	76.80
ReBiDet * [28]	86.23	85.89	**61.99**	82.41	**67.86**	**83.94**	89.78	**90.88**	**86.37**	**83.70**	**72.12**	**77.58**	**78.38**	73.24	75.01	52.05	77.96
RTMDet-R-l * [43]	**89.31**	**86.38**	55.09	**83.17**	66.11	82.44	**89.85**	90.84	**86.95**	**83.76**	68.35	74.36	77.60	**77.39**	**77.87**	**60.37**	**78.12**
ReBiDet * + ${\mathrm{SA}}^{3}$	**88.76**	86.35	60.97	81.93	**73.39**	**84.26**	**90.05**	**90.88**	**87.20**	83.30	**72.19**	**77.07**	**78.67**	72.62	72.94	**55.34**	**78.50**
ReBiDet * + ${\mathrm{SA}}^{3}$ + EAS	86.84	85.58	**62.23**	**82.60**	**68.05**	**83.92**	89.83	**90.90**	86.91	83.23	**73.62**	**76.64**	**78.54**	72.12	**77.53**	**63.05**	**78.85**

* indicates multi-scale training and testing. Note. The RetinaNet OBB (RetinaNet-O) [40], Faster R-CNN OBB (FR-O) [37], Mask R-CNN [30], and Hybrid Task Cascade (HTC) [29] results are based on a reproduced version of DOTA-v1.5 [38] and have been used by some scholars [23,43]. “Single-scale” indicates the model is directly trained and tested on the DOTA-v1.5 dataset, while “multi-scale” indicates the model is trained and tested using random rotation and offline multi-scale enhancement on DOTA-v1.5. For ease of reading and comparison, the first, second, and third highest values in each column are marked in red, yellow, and green, respectively, and are bolded.

## Data Availability

The DOTA dataset was publicly released by Wuhan University in November 2017 and includes complete sets of training, validation, and testing images, as well as annotation files for the training and validation sets. It can be downloaded at this address: https://captain-whu.github.io/DOTA/dataset.html (accessed on 29 May 2024). Since the downloaded package does not include annotation files for the testing set, users need to submit their model’s inference results to the Wuhan University official Evaluation Server to obtain the accuracy of the inference results, at this address: https://captain-whu.github.io/DOTA/evaluation.html (accessed on 29 May 2024). After this article is published, our code and other related materials will also be released.

## Abbreviations

The following abbreviations are used in this manuscript:

IoU	Intersection over Union
${\mathrm{SA}}^{3}$	Spatial Adaptive Angle-Aware
EAS Loss	Edge-aware Skewed Bounding Box Loss
RPN	Region Proposal Network
